# The “case” for case studies: why we need high-quality examples of global implementation research

**DOI:** 10.1186/s43058-021-00227-5

**Published:** 2022-02-16

**Authors:** Blythe Beecroft, Rachel Sturke, Gila Neta, Rohit Ramaswamy

**Affiliations:** 1 Fogarty International Center, US National Institutes of Health, Bethesda, USA; 2 National Cancer Institute, US National Institutes of Health, Bethesda, USA; 3 Cincinnati Children’s Hospital Medical Center, Cincinnati, USA

**Keywords:** Case studies, Global implementation science, LMICs

## Abstract

Rigorous and systematic documented examples of implementation research in global contexts can be a valuable resource and help build research capacity. In the context of low- and middle-income countries (LMICs), there is a need for practical examples of how to conduct implementation studies. To address this gap, Fogarty’s Center for Global Health Studies in collaboration with the Cincinnati Children's Hospital Medical Center and the National Cancer Institute is commissioning a collection of implementation science case studies in LMICs that describe key components of conducting implementation research, including how to select, adapt, and apply implementation science models, theories, and frameworks to these settings; develop and test implementation strategies; and evaluate implementation processes and outcomes. The case studies describe implementation research in various disease areas in LMICs around the world. This commentary highlights the value of case study methods commonly used in law and business schools as a source of “thick” (i.e., context-rich) description and a teaching tool for global implementation researchers. It addresses the independent merit of case studies as an evaluation approach for disseminating high-quality research in a format that is useful to a broad range of stakeholders. This commentary finally describes an approach for developing high-quality case studies of global implementation research, in order to be of value to a broad audience of researchers and practitioners.

Contributions to the literature

- Reinforcing the need for “thick” (i.e., context-rich) descriptions of implementation studies
- Highlighting the utility of case studies as a dissemination strategy for researchers, practitioners, and policymakers
- Articulating the value of detailed case studies as a teaching tool for global implementation researchers
- Describing a method for developing high-quality case studies of global implementation research

## Background

Research capacity for implementation science remains limited in low- and middle-income countries (LMICs). Various stakeholders, including NIH-funded implementation researchers and practitioners, often inquire about how to apply implementation science methods and have requested additional resources and training to support implementation capacity building. This is in part due to a dearth of practical examples for both researchers and practitioners of how to select, adapt, and apply implementation science models, theories, and frameworks to these settings; how to evaluate implementation processes and outcomes; and how to develop and test implementation strategies. The need for detailed documentation of implementation research in all settings has been well established, and guidelines for documentation of implementation research studies have been created [1, 2]. But the mere availability of checklists and guidelines in and of themselves does not result in comprehensive documentation that is useful for learning, as has been pointed out by many systematic reviews of implementation science and quality improvement studies ([3, 4]). It has also been observed that documentation alone is not enough, and there is a need for mentors to translate abstract theories into context-appropriate research designs and practice approaches [5]. Because of the especially acute shortage of mentors and coaches in LMIC settings, we propose that documentation with “thick” descriptions that go beyond checklists and guidelines are needed to make the field more useful to emerging professionals [6]. We suggest that the case study method intended to “explore the space between the world of theory and the experience of practice” [7] that has been used successfully for over a century by law and business schools as a teaching aid can be of value to develop detailed narratives of implementation research projects. In this definition, we are not referring to the case study as a qualitative research method [8], but as a rich and detailed method of retrospective documentation to aid teaching, practice, and research. In this context, our case studies are akin to “single-institution or single-patient descriptions” [9] called “case reports” or “case examples” in other fields. As these terms are rarely used in global health, we have used the words “case studies” in this paper but reiterate that they do not refer to case study research designs.

Fogarty’s Center for Global Health Studies (CGHS) in collaboration with the Cincinnati Children's Hospital Medical Center and the National Cancer Institute (NCI) is commissioning a collection of implementation science case studies that describe implementation research focusing on various disease areas in different (LMIC) contexts around the world. These case study descriptions will provide guidance on the process of conducting implementation science studies and will highlight the impact these studies have had on practice and policy in global health contexts. This brief note makes a case for using case studies to document and disseminate implementation research, describes the CGHS approach to case study development and poses evaluation questions that need to be answered to better understand the utility of case studies. This effort is intended to develop a set of useful examples for LMIC researchers, practitioners, and policymakers, but also to assess and improve the use of case studies as a tested documentation methodology in implementation research.

## Main text

### The “case” for case studies

A preliminary landscape analysis of the field conducted by CGHS found that there are not many descriptions of global implementation science projects in a case study format in the peer-reviewed or gray literature, and those that exist are embedded in the content of academic teaching materials. There is not a cohesive collection, especially relating to health, that illustrates how implementation research has been conducted in varied organizations, countries, or disease areas. This new collection will add value in three different ways: as a dissemination strategy, as a tool for capacity building, and as a vehicle for stimulating better research.

### Case studies as a dissemination strategy

Case studies have independent merit as an evaluation approach for disseminating high-quality research in a format that is useful to a broad range of stakeholders. The Medical Research Council (MRC) has recommended process evaluation as a useful approach to examine complex implementation, mechanisms of impact, and context [10]. Guidelines on documentation of implementation recommend that researchers should provide “detailed descriptions of interventions (and implementation strategies) in published papers, clarify assumed change processes and design principles, provide access to manuals and protocols that provide information about the clinical interventions or implementation strategies, and give detailed descriptions of active control conditions” [1]. Case studies can be thought of as a form of post hoc process evaluation, to disseminate *how* the delivery of an intervention is achieved, the mechanisms by which implementation strategies produce change, or how context impacts implementation and related outcomes.

### Case studies as a capacity building tool

In addition, case studies can address the universal recognition of the need for more capacity building in *Implementation Science*, especially in LMIC settings. Case studies have been shown to address common pedagogical challenges in helping students learn by allowing students to dissect and explore limitations, adaptations, and utilization of theories, thereby creating a bridge between theories presented in a classroom and their application in the field [11]. A recent learning needs assessment for implementation researchers, practitioners, and policymakers in LMICs conducted by Turner et al. [12] reflected a universal consensus on the need for context-specific knowledge about how to apply implementation science in practice, delivered in an interactive format supported by mentorship. A collection of case studies is a valuable and scalable resource to meet this need.

### Using case studies to strengthen implementation research

Descriptions of research using studies can illustrate not just whether implementation research had an impact on practice and policy, but how, why, under what circumstances, and to whom, which is the ultimate goal of generating generalizable knowledge about how to implement. Using diverse cases to demonstrate how a variety of research designs have been used to answer complex implementation questions provides researchers with a palette of design options and examples of their use. A framework developed by Minary et al. [13] illustrates the wide variety of research designs that are useful for complex interventions, depending on whether the emphasis is on internal and external validity or whether knowledge about content and process or about outcomes is more important. A collection of case studies would be invaluable to researchers seeking to develop appropriate designs for their work. In addition, the detailed documentation provided through these case descriptions will hopefully motivate researchers to document their own studies better using the guidelines described earlier.

### Developing and testing the case study creation process: the CGHS approach

Writing case studies that satisfy the objectives described above is an implementation science undertaking in itself that requires the engagement of a variety of stakeholders and planned implementation strategies. The CGHS team responsible for commissioning the case studies began this process in 2017 and followed the approach detailed below to test the process of case study development.

1. Conducted 25+ consultations with various implementation science experts on gaps in the field and the relevance of global case studies
2. Convened a 15-member Steering Committee^1^ of implementation scientists with diverse expertise, from various academic institutions and NIH institutes to serve as technical experts and to help guide the development and execution of the project
3. Developed a case study protocol in partnership with the Steering Committee to guide the inclusion of key elements in the case studies
4. Commissioned two pilot cases^2^ to assess the feasibility and utility of the case study protocol and elicited feedback on the writing experience and how it could be improved as the collection expands
5. Led an iterative pilot writing process where each case study writing team developed several drafts, which were reviewed by CGHS staff and a designated member of the Steering Committee
6. Truncated and adjusted the protocol in response to input from the pilot case study authors teams
7. Developed a comprehensive grid with the Steering Committee, outlining the key dimensions of implementation science that are significant and would be important areas of focus for future case studies. The grid will be used to evaluate potential case applicants and is intended to help foster diversity of focus and content, in addition to geography

### Implementing the process: the call for case studies

In March of 2021, CGHS issued a closed call for case studies to solicit applications from a pool of researchers. Potential applicants completed the comprehensive grid in addition to a case study proposal. Applicants will go through a three-tier screening and review process. CGHS will initially screen the applications for completeness to ensure all required elements are present. Each case study application will then be reviewed by two Steering Committee members for content and scientific rigor and given a numerical score based on the selection criteria. Finally, the CGHS team will screen the applications to ensure diversity of implementation elements, geography, and disease area. Approximately 10 case studies will be selected for development in an iterative process. Each case team will present their case drafts to the Steering Committee, which will collectively workshop the drafts in multiple sittings, drawing on the committee’s implementation science expertise. Once case study manuscripts are accepted by the Steering Committee, they will be submitted to *Implementation Science Communications* for independent review by the journal. CGHS intends for the case studies to be published collectively, but on a rolling basis as they are accepted for publication.

### Future research: evaluating the effectiveness of the case study approach

This commentary has put forth arguments for the potential value of case studies for documenting implementation research for researchers, practitioners, and policymakers. Case studies not only provide a way to underscore how implementation science can advance practice and policy in LMICs, but also offer guidance on how to conduct implementation research tailored to global contexts. However, there is little empirical evidence about the validity of these arguments. The creation of this body of case studies will allow us to study why, how, how often, and by whom these case studies are used. This is a valuable opportunity to learn and use that information to better inform future use of this approach as a capacity-building or dissemination strategy.

## Conclusions

Similar to their use in law and business, case study descriptions of implementation research could be an important mechanism to counteract the paucity of training programs and mentors to meet the demands of global health researchers. If the evaluation results indicate that the case study creation process produces useful products that enhance learning to improve future implementation research, a mechanism needs to be put in place to create more case studies than the small set that can be generated through this initiative. There will be a need to create a set of documentation guidelines that complement those that currently exist and a mechanism to solicit, review, publish, and disseminate case studies from a wide variety of researchers and practitioners. Journals such as *Implementation Science* or *Implementation Science Communications* can facilitate this effort by either creating a new article type or by considering a new journal with a focus on rigorous and systematic case study descriptions of implementation research and practice. An example that could serve as a guide is *BMJ Open Quality*, which is a peer-reviewed, open-access journal focused on healthcare improvement. In addition to original research and systematic reviews, the journal publishes two article types: *Quality Improvement Report* and *Quality Education Report* to document healthcare quality improvement programs and training. The journal offers resources for authors to document their work rigorously. Recently, a new journal titled *BMJ Open Quality South Asia* has been released to disseminate regional research. We hope that our efforts in sponsoring and publishing these cases, and in setting up a process to support their creation, will make an important contribution to the field and become a mechanism for sharing knowledge that accelerates the growth of implementation science in LMIC settings.

## Data Availability

Not applicable.

## Abbreviations

LMICs: Low- and middle-income countries
CGHS: Center for Global Health Studies
NCI: National Cancer Institute
MRC: Medical Research Council

## Footnotes

1 Rohit Ramaswamy, CCHMC, Gila Neta, NCI NIH, Theresa Betancourt, BC, Ross Brownson, WASU, David Chambers, NCI NIH, Sharon Straus, University of Toronto, Greg Aarons, UCSD, Bryan Weiner, UW, Sonia Lee, NICHD NIH, Andrea Horvath Marques, NIMH NIH, Susannah Allison, NIMH NIH, Suzy Pollard, NIMH NIH, Chris Gordon, NIMH NIH, Kenny Sherr, UW, Usman Hamdani, HDR Foundation Pakistan, Linda Kupfer, FIC NIH

2 The first pilot case was led by the Human Development Research Foundation (HDRF) in Pakistan and examines scaling up evidenced-based care for children with developmental disorders in rural Pakistan. The second pilot was led by Boston College and investigates alternate delivery platforms and implementation models for bringing evidence-based behavioral Interventions to scale for youth facing adversity in Sierra Leone to close the mental health treatment gap.

## References

[1] Proctor EK, Powell BJ, McMillen JC. Implementation strategies: recommendations for specifying and reporting. Implementation Sci. 2013;8(139) [cited 2021 May];. Available from: https://implementationscience.biomedcentral.com/articles/10.1186/1748-5908-8-139. 10.1186/1748-5908-8-139 PMC3882890 24289295

[2] Pinnock H, Barwick M, Carpenter CR, Eldridge S, Grandes G, Griffiths CJ (2017). for the StaRI group. Standards for Reporting Implementation Studies (StaRI) statement. BMJ.

[3] Brouwers MC, De Vito C, Bahirathan L, Carol A, Carroll JC, Cotterchio M, et al. What implementation interventions increase cancer screening rates? A systematic review. Implementation Sci. 2011; [cited 2021 Sept];6:111. Available from: https://www.ncbi.nlm.nih.gov/pmc/articles/PMC3197548/. 10.1186/1748-5908-6-111 PMC3197548 21958556

[4] Nadeem E, Gleacher A, Beidas RS. Consultation as an implementation strategy for evidence-based practices across multiple contexts: unpacking the black box. Adm Policy Ment Health. 2013;40(6):439–50. [cited 2021 Sept]. Available from: https://link.springer.com/article/10.1007%2Fs10488-013-0502-8. 10.1007/s10488-013-0502-8 PMC3795855 23716145

[5] Darnell D, Dorsey CN, Melvin A, Chi J, Lyon AR, Lewis CC (2017). A content analysis of dissemination and implementation science resource initiatives: what types of resources do they offer to advance the field?. Implement Sci.

[6] Geertz C. Thick description: toward an interpretive theory of culture In: The interpretation of cultures: selected essays. New York: Basic Books; 1973. p. 3–32.

[7] Breslin M, Buchanan R (2008). On the case study method of research and teaching in design. Design Issues.

[8] Crowe S, Cresswell K, Robertson A, Huby G, Avery A, Sheikh A (2011). The case study approach. BMC Med Res Methodol.

[9] Alpi KM, Evans JJ (2019). Distinguishing case study as a research method from case reports as a publication type. J Med Libr Assoc.

[10] Moore GF, Audrey S, Barker M, Bond L, Bonell C, Hardeman W (2015). Process evaluation of complex interventions: Medical Research Council guidance. BMJ.

[11] Velenchik AD (1995). The case method as a strategy for teaching policy analysis to undergraduates. J. Econ. Educ..

[12] Turner MW, Bogdewic S, Agha E, Blanchard C, Sturke R, Pettifor A, et al. Learning needs assessment for multi-stakeholder implementation science training in LMIC settings: findings and recommendations. Implement Sci Commun. 2021;2(134) [cited 2021 May]. Available from: https://implementationsciencecomms.biomedcentral.com/articles/10.1186/s43058-021-00238-2. 10.1186/s43058-021-00238-2 PMC8642989 34863314

[13] Minary L, Trompette J, Kivits J, Cambon L, Tarquinio C, Alla F. Which design to evaluate complex interventions? Toward a methodological framework through a systematic review. BMC Med Res Methodol. 2019;19(92) [cited 2021 May]. Available from: https://bmcmedresmethodol.biomedcentral.com/articles/10.1186/s12874-019-0736-6. 10.1186/s12874-019-0736-6 PMC6505260 31064323

